# Graphene plasmons-enhanced terahertz response assisted by metallic gratings

**DOI:** 10.1515/nanoph-2022-0455

**Published:** 2022-11-04

**Authors:** Anqi Yu, Zhenyu Yang, Miao Cai, Huiping Zhang, Zhengan Tian, Xuguang Guo, Lanxia Wang, Alexei V. Balakin, Alexander P. Shkurinov, YiMing Zhu

**Affiliations:** Shanghai Key Lab of Modern Optical System, Terahertz Technology Innovation Research Institute, Terahertz Spectrum and Imaging Technology Cooperative Innovation Center, University of Shanghai for Science and Technology, 516 Jungong Road, Shanghai 200093, China; Shanghai Institute of Intelligent Science and Technology, Tongji University, Shanghai 200092, China; Shanghai International Travel Health Care Center (Shanghai Customs Port Clinic), 2090 Jinqiao Road, Shanghai 200125, China; Faculty of Physics and International laser Center, Lomonosov Moscow State University, Leninskie Gory 1-2, Moscow 19991 Russia; ILIT RAS – Branch of the FSRC “Crystallography and Photonics” RAS, Svyatoozerskaya 1, 140700, Shatura, Moscow Region, Russia

**Keywords:** graphene plasmons, metallic gratings, photo-thermoelectric effect, plasmon rectification effect, terahertz detector

## Abstract

Terahertz detectors based on two-dimensional Dirac materials offer a new approach for room-temperature terahertz detection with high response and low noise. However, these devices can hardly show high response over a broad frequency range, mainly due to the poor absorption caused by their ultrathin nature. Here we apply metallic gratings to enhance the excitation efficiency of graphene plasmons. When nonzero source-drain bias is applied, graphene plasmons can generate terahertz response orthogonal to the polarization of the incidence. The response is attributed to the orthogonal overdamped plasmon rectification effect, and graphene plasmons-enhanced photo-thermoelectric effect. By comparing the normalized on/off ratio, the metallic gratings are found to effectively enhance the coupling efficiency between graphene plasmons and THz incidence, and thus the absorption and responsivity. The results are beneficial for improving the response of room temperature THz detectors.

## 1 Introduction

Terahertz (THz) technology has attracted a lot of interest in recent years because of its potential applications in biomedicine [1–3], security [4–6] and communication [7–9]. Many of these applications require operations at room temperature with high efficiency. As a result, room-temperature THz detectors with high responsivity, low noise equivalent power (NEP), and broadband response are desired. However, electronic THz detectors generally suffer from low responsivity or narrow bandwidth because the relaxation time of charge carriers is longer than a period of THz wave, while photonic THz detectors generally require cryogenic environment because of the low single photon energy.

In recent years, two-dimensional materials with Dirac band structure are receiving more and more attention in THz detection for that electron-phonon interaction is quite weak in these materials [10]. THz detectors based on these materials show low NEP at room temperature. Cai et al. demonstrated room-temperature detection via the photo-thermoelectric (PTE) effect in graphene and achieved peak responsivity of 715 V/W with NEP less than 20 pW/Hz^1/2^ at 2.52 THz [11]. Castilla et al. designed an antenna-integrated graphene THz detector and realized detection from 1.8–4.25 THz with peak responsivity of ∼105 V/W and NEP of 80 pW/Hz^1/2^ [12]. Guo et al. used type II Dirac semimetal PdTe_2_ as the detector channel and achieved responsivity of 0.2(/10) A/W at 0(/0.1) V source–drain bias with NEP of ∼1(/53) pW/Hz^1/2^ at 0.12(/0.3) THz [13]. Bandurin et al. fabricated a graphene field effect transistor and found maximum responsivity of 20 V/W and minimum NEP of 600 pW/Hz^1/2^ [14]. All of the above-mentioned data are measured at room temperature, exhibiting NEP from tens to hundreds pW/Hz^1/2^. Nevertheless, the responsivities of these devices can hardly achieve 1000 V/W, which results from the limited absorption of these few-layer two-dimensional materials. Increasing the material thickness (layer number) will help to enhance the absorption. However, the advantages of the two-dimensional materials will possibly be lost (e.g., the massless nature of electrons and extremely high mobility in monolayer graphene no longer exist for few-layer graphene). Increasing the carrier density can also enhance the absorption, but cannot guarantee higher response. For the Dyakonov–Shur rectification effect and the PTE effect, Ref. [14] showed that the responsivity will reduce if the gate voltage keeps going up. For other THz detection mechanisms, e.g., plasmon drag effect and plasmon ratchet effect, Refs. [15, 16] showed that the responsivity will also decrease as the charge carrier density increases. Therefore, increasing absorption with limited layer number and carrier density is key to higher response for these THz detection mechanisms.

In this work, we apply metallic gratings to enhance the absorption of graphene micro-ribbons. The simulated spectra show that the absorption is enhanced by at least 4 times within 1 THz for that graphene plasmons interact with the spoof plasmons of metallic gratings. Then the simulated structure is fabricated and the THz response is measured. Compared with graphene micro-ribbons without metallic gratings, graphene micro-ribbons with metallic gratings show stronger THz response when the polarization of incidence is perpendicular to the gratings, and the difference increases as the incidence frequency increases from 0.04 THz to 0.6 THz. Then it is concluded that metallic gratings can help to enhance the absorption of graphene plasmons to the THz incidence, resulting in higher THz response. The polarization dependence and the response time suggest that there are two mechanisms responsible for the response. We attributed the fast one to the orthogonal plasmon rectification effect, and the slow one to the plasmons-enhanced PTE effect. These results are helpful for enhancing the THz response of PTE or plasmonic THz detectors, offering an approach for realizing room-temperature broadband THz detector with high responsivity.

## 2 Simulation

**Figure 1:**
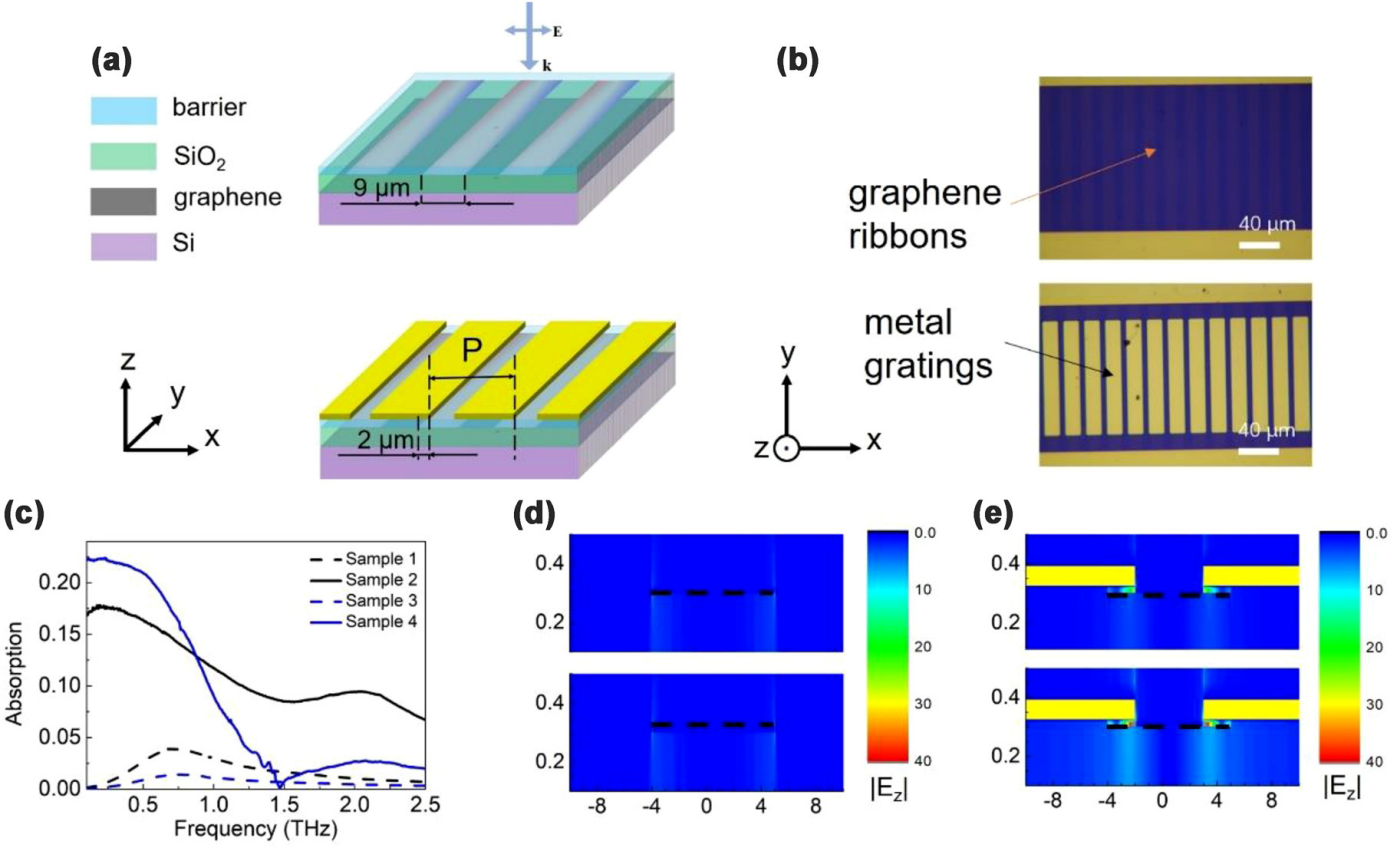
The designed structure, their absorption spectra and |E_z_| distribution. (a) The simulated graphene micro-ribbon structure without (upper panel) and with (lower panel) metallic gratings. (b) The microscopic images of Sample 1 (upper panel) and Sample 2 (lower panel). (c) The absorption spectra for Sample 1 (black dashed curve), Sample 2 (black solid curve), Sample 3 (blue dashed curve) and Sample 4 (blue solid curve). (d) The |E_z_| distribution of Sample 1 (upper panel) and Sample 3 (lower panel) at 0.6 THz, with graphene indicated by black dashed lines. (e) The |E_z_| distribution of Sample 2 (upper panel) and Sample 4 (lower panel) at 0.6 THz, with graphene indicated by black dashed lines and metal indicated by golden squares.

In our previous work, we numerically demonstrated that plasmons excited in sparse periodic graphene micro-squares can be enhanced by metallic split-mesh [17]. In this work, we design a one-dimensional counterpart, that is, graphene micro-ribbons with metallic gratings. In order that the graphene plasmons can be excited around 1 THz, the width of the graphene micro-ribbons is assumed to be 9 μm along the *x*-coordinate. The length is assumed to be infinite along the *y*-coordinate, as schematically shown in the upper panel of Figure 1(a). The thickness of graphene is assumed to be 1 nm. The minimum mesh is 0.1 nm around graphene for *z*-coordinate and 1 nm for *x*- and *y*-coordinates. Graphene is characterized by the Kubo formula [18, 19], with Fermi level assumed to be 0.05 eV. Graphene ribbons are supported by a Si/SiO_2_ substrate. The thickness of Si is assumed to be semi-infinite and the refractive index is assumed to be 3.4. The thickness of SiO_2_ is assumed to be 300 nm and the refractive index is assumed to be 1.7. An ultrathin dielectric layer on top of graphene ribbons is used to separate the graphene ribbons and the metallic gratings, such that graphene plasmons can be excited at lower frequencies because metal in close vicinity of semiconductors can change the dispersion relation of plasmons [20]. The increase in thickness or the decrease in refractive index of the ultrathin dielectric layer will cause the blueshift of graphene plasmons. In order that the enhanced plasmonic absorption covers the frequencies below 1 THz, the thickness of the ultrathin dielectric layer is chosen to be 30 nm and the refractive index is 1.7. Metal is characterized as perfect electric conductor. The width of the metallic gratings is (*P* − 5) μm, where *P* is the width of a unit cell. The width of the overlap region between the metallic gratings and graphene ribbons is 2 μm for both sides. It should be pointed out that, we temporarily neglect the absorption of Si and characterize graphene with a high scattering time of 0.2 ps to better study the enhancement of graphene plasmons.

Firstly, we numerically study the proposed structure by finite-difference-time-domain (FDTD) simulation, with the polarization of incidence perpendicular to the graphene micro-ribbons. Four structures are simulated: *P* = 20 μm without metallic gratings (labelled as Sample 1), *P* = 20 μm with metallic gratings (as shown in the lower panel of Figure 1(a), labelled as Sample 2), *P* = 60 μm without metallic gratings (labelled as Sample 3), and *P* = 60 μm with metallic gratings (labelled as Sample 4). As a result of metallic gratings-enhanced coupling efficiency between graphene plasmons and the incident THz waves, Sample 2 and Sample 4 show stronger absorption than Sample 1 and Sample 3, as shown in Figure 1(c). Meanwhile, enhanced coupling efficiency will lead to larger radiative damping, such that the resonant peaks are broadened. Similar to the results in our previous work [17], the difference in absorption between Sample 4 and Sample 3 is larger than that between Sample 2 and Sample 1, which means that the proposed structure is more efficient when the duty cycle of graphene is lower. Figure 1(d) shows the |E_z_| distribution of Sample 1 and Sample 3 and Figure 1(e) shows the |E_z_| distribution of Sample 2 and Sample 4 at 0.6 THz. Obviously, Sample 2 and Sample 4 show stronger |E_z_|, which is an indication of enhanced excitation efficiency of graphene plasmons. Because the duty cycle of graphene in Sample 4 is 3 times lower than that in Sample 2 and the absorption of the former is slightly higher, the |E_z_| distribution in Sample 4 is stronger than that in Sample 2. Intuitively, stronger |E_z_| distribution will lead to stronger THz absorption in a single graphene ribbon, yielding responses with higher on/off ratio.

## 3 Experiment

Figure 1(b) gives the microscopy images of Sample 1 (upper panel) and Sample 2 (lower panel). After fabrication, the transfer characteristic curve of the devices was tested by Agilent B2912. Both Sample 1 and Sample 3 show typical V-shape curves which increases sub-linearly as the back-gate voltage increases, as shown in Figure S1(a) and (c). The mobility and Fermi level of Sample 1 and Sample 3 as a function of back-gate voltage are shown in Figure S1(b) and (d). It is seen that the mobility of Sample 1 and Sample 3 peaks at about 2600 cm^2^/Vs and drops as the absolute value of the back-gate voltage increases. The Fermi level increases from ∼0.04 eV to ∼0.11 eV. The anomaly around 0 V in Figure S1(b) and (d) is the result of the tilted Fermi level along the channel, which is explained in detail in Figure S2. Figure S1(e), (f) and (h) shows that the mobility and the carrier relaxation time of Sample 1 and Sample 3 drop a little after six months. Comparatively, Sample 2 do not show such sharp V-shape curves, as shown in Figure S2(g), possibly due to the non-uniform effective capacitance between the gated graphene and the ungated graphene as shown in the inset of Figure S2(g).

**Figure 2:**
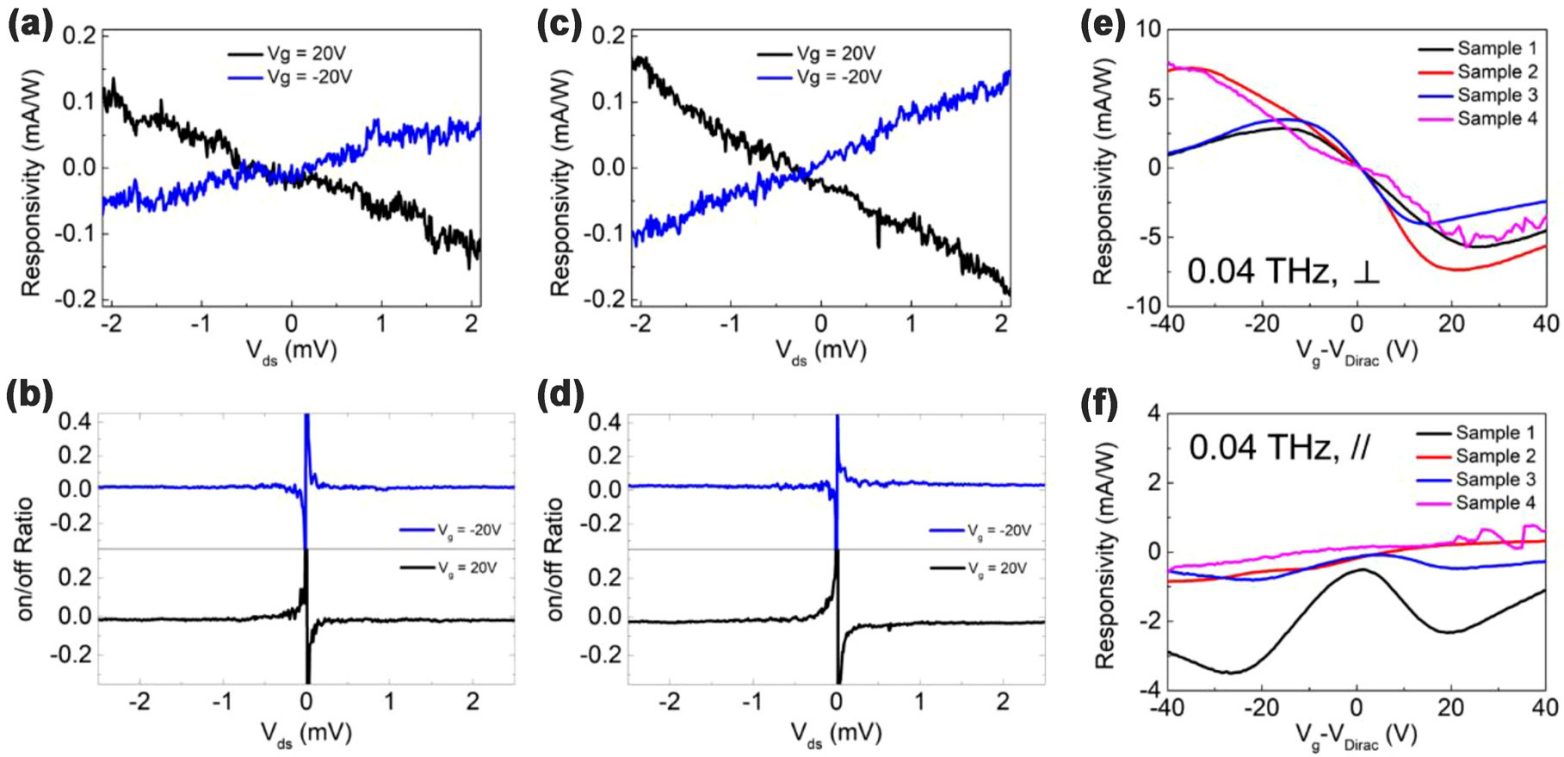
The responsivity and on/off ratio under 0.04 THz illumination. (a) The responsivity of Sample 1 as a function of source-drain bias with the gate voltage fixed at 20 V and −20 V, respectively. (b) The on/off ratio corresponding to (a). (c) The responsivity of Sample 2 as a function of source–drain bias with the gate voltage fixed at 20 V and −20 V, respectively. (d) The on/off ratio corresponding to (c). (e) The responsivity of the 4 devices as a function of gate voltage with source-drain bias fixed at 0.1 V, and the incidence polarization perpendicular to the graphene ribbons. (f) The responsivity of the 4 devices as a function of gate voltage with source-drain bias fixed at 0.1 V, and the incidence polarization parallel to the graphene ribbons.

**Figure 3:**
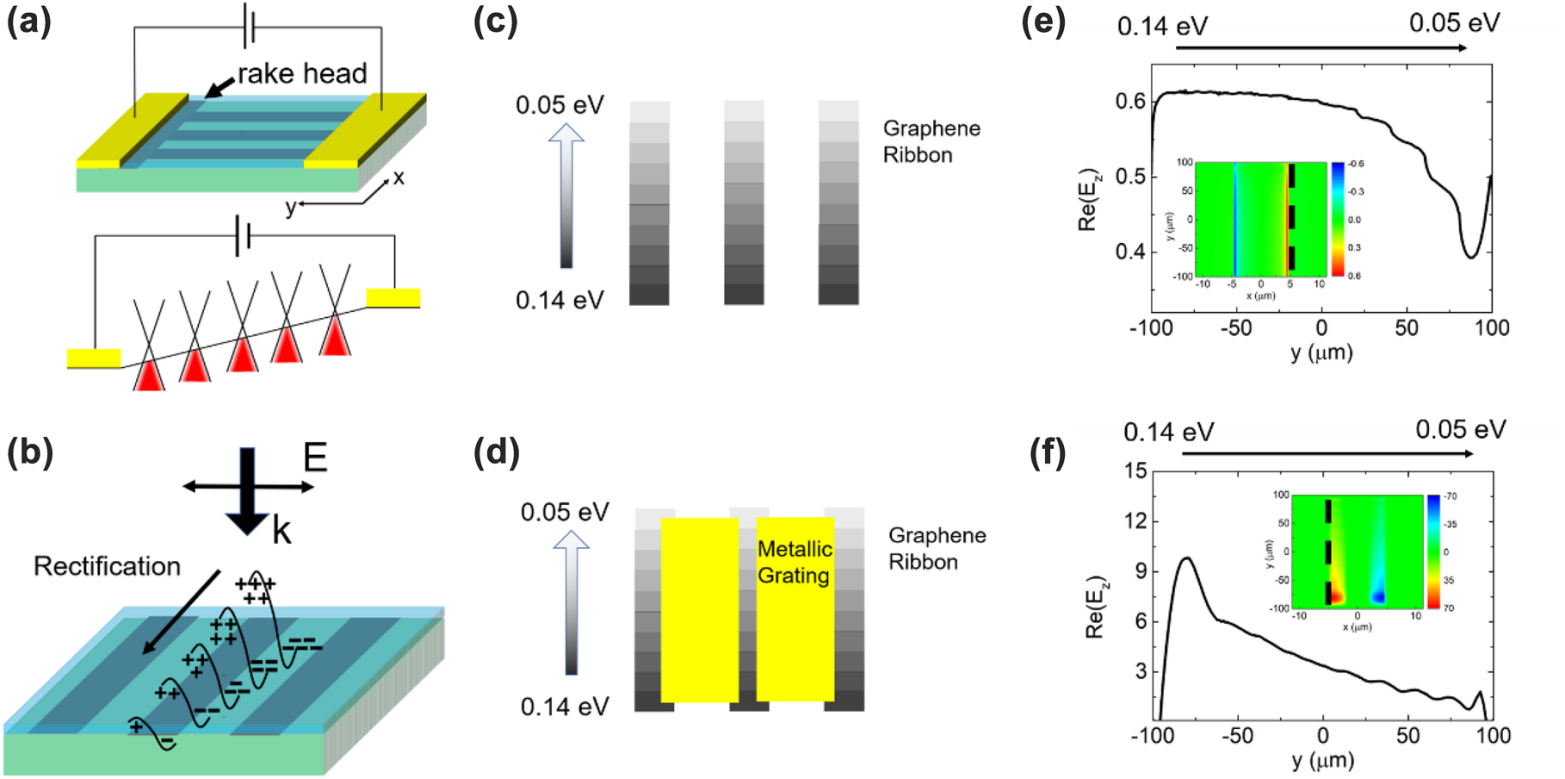
Illustration of bias-induced asymmetry and rectification. (a) Illustration of the tilted energy band when a positive source-drain bias is applied. (b) Schematics for the change in the strength of plasmon resonances and the rectification effect. (c) Schematics of simulating Sample 1 with gradient Fermi level, in which each graphene ribbon is divided into 10 parts with 0.01 eV a step. (d) Schematics of the simulating Sample 2 with gradient Fermi level, in which each graphene ribbon is divided into 10 parts with 0.01 eV a step. (e) The simulated Re(E_z_) distribution of Sample 1 along the black dashed in the inset by separating the graphene ribbon into 10 parts. (f) The simulated Re(E_z_) distribution of Sample 2 along the black dashed in the inset by separating the graphene ribbon into 10 parts.

The fabricated devices were then illuminated by a 40 GHz microwave source with the source-drain bias swept from −2 mV to 2 mV and the gate kept at 20 V and −20 V, respectively. Figure 2(a) shows the response and Figure 2(b) shows the on/off ratio of Sample 1. Here the on/off ratio is calculated by *ΔI*/*I*_off_, where *ΔI* represents the change in measured current when the source is turned on, and I_off_ represents the measured current when the source is turned off. The response and the on/off ratio of Sample 2 is shown in Figure 2(c) and (d). It is seen that the response changes linearly as the source–drain bias changes. The on/off ratio is nearly constant when the source-drain bias is away from 0, which is an indication of photoconductive-like mechanism. When the source-drain bias is extremely close to 0, the on/off ratio increases/drops suddenly, which is an indication of photovoltage-like mechanism. Thus, the response can result from a combination of a photovoltage-like mechanism and a photoconductive-like mechanism. Besides, photovoltage-like mechanisms such as photo thermo-electric (PTE) and plasmon rectification effect can also be the only contributor because they show 0-bias response and the response changes as the source-drain bias changes. The PTE effect can often be observed because of the asymmetry brought by the misalignment in the fabrication process, generating a zero-bias signal (photovoltage-like). Once biased, the Seebeck coefficient across the channel will change accordingly, and then the photocurrent will also change (photoconductive-like) [21]. For the plasmon rectification effect, the weak fabrication misalignment can also induce in the difference of localized electric field, and thus a zero-bias signal (photovoltage-like). When a nonzero source-drain bias is applied, the localized electric field strengths and the wavevector for the forward- and backward-plasmon waves deviate from each other as the source-drain bias increases, resulting in a photoconductive-like response (photoconductive-like) [22, 23]. It should be pointed out that, apart from the unavoidable fabrication misalignment, the rake head shape of graphene as indicated in Figure 3(a) in our devices also brings asymmetry, which can also be the source of the response at zero bias.

In order to identify the mechanism of the THz response, the response as a function of gate voltage (with the polarization of incidence perpendicular to the graphene ribbons) is measured with source-drain bias fixed at 0.01 V. The shape of the response as shown in Figure 2(e) is quite similar to that given in Ref. [14]. In Ref. [14] the response was attributed to the PTE effect and the plasma wave rectification effect at different gate voltage. For our devices, if the pure PTE effect is the main contributor to the response, the shape of response should be polarization-independent. Then the polarization of incidence is rotated by 90° and the corresponding response is shown in Figure 2(f). Comparing Figure 2(e) and (f), one can see that the shapes of response under the two orthogonal polarizations are quite different, which means that pure PTE effect is unlikely to be the main contributor to the response.

In Type-II Dirac materials, polarization-dependent response can be generated due to the asymmetric scattering of charge carriers brought by lattice asymmetry [13, 24]. However, graphene does not possess such lattice asymmetry. Because the polarization-dependence in response exists whether the metallic gratings exist or not, the polarization-dependence should be decided by the graphene micro-ribbons rather than the metallic gratings. Note that the response of our devices shows peaking profile as shown in Figure 2(e), similar to the response of plasma wave rather than the response of the PTE effect as given in Ref. [14]. Since the extracted graphene carrier relaxation time *τ* is ∼13 fs for electrons and ∼9 fs for holes, as shown in Figure S1(h), the excited graphene plasmons must be overdamped because *ωτ* << 1. Then it can be inferred that the polarization-dependent response is related to overdamped graphene plasmons. Previously, photovoltage-like response of the plasmon rectification effect relying on the asymmetric distribution of localized electric field was both theoretically analyzed and experimentally demonstrated [16, 23, 25], [26], [27], [28]. Photoconductive-like response of plasmons under nonzero source-drain bias was also studied [22, 23]. In these works, the response was parallel to the polarization of the incidence. To the best of our knowledge, plasmonic response perpendicular to the polarization of the incidence has not been reported. Generally, in the direction perpendicular to the polarization of incidence, the strength of localized electric field is the same if the applied bias is 0, so that there will be no rectification-caused photo-response. When the source-drain bias is nonzero, the Fermi level along the graphene ribbon will change gradually along the y-coordinate, as schematically shown in Figure 3(a). Consequently, the excitation efficiency of plasmons can be different along the y-coordinate, which will induce in a tilted localized electric field *E*_*z*_ along the *y*-coordinate, as schematically shown in Figure 3(b). Nonzero ∇*E*_*z*_(*y*,*t*) will lead to nonzero Δ*E*_*y*_(*y*,*t*), which will lead to harmonic perturbation in carrier velocity Δ*v*(*y*,*t*). Besides, nonzero ∇(*E*_*z*_) itself is an indication of harmonic perturbation in carrier density Δ*n*(*y*,*t*). That is to say, the *E*_*z*_ distribution along the *y*-coordinate in our devices can be considered as an analogue to that in single gate high electron mobility transistors, generating photoconductive-like THz response along the channel.

To verify the above conjecture, FDTD simulation is performed by varying the Fermi level of graphene from source to drain. In order to show the difference in Re(*E*_*z*_) more clearly, the difference in Fermi level was set to vary from 0.05 eV to 0.14 eV with 0.01 eV a step, as shown in Figure 3(c), and the relaxation time was set as 10 times the value retrieved from the measured data shown in Figure S1(h). The inset of Figure 3(e) shows that Re(*E*_*z*_) is slightly stronger at the low Fermi level side and reduces monotonically along the y-coordinate for Sample 1. The linear cut of Re(*E*_*z*_) along the black dashed line shows that Re(*E*_*z*_) reduces steplike at the boundary between two adjacent graphene regions. The sudden drop at the boundary between two graphene blocks comes from the steplike change in Fermi level. In realistic devices, where Fermi level changes gradually, the change in Re(*E*_*z*_) should be smooth and gradual and there should be no sudden drop. Figure 3(f) shows similar change in Re(*E*_*z*_) for Sample 2, but the magnitude is much higher than that shown in Figure 3(e), which is the reason that Sample 2 shows stronger response than Sample 1 does.

**Figure 4:**
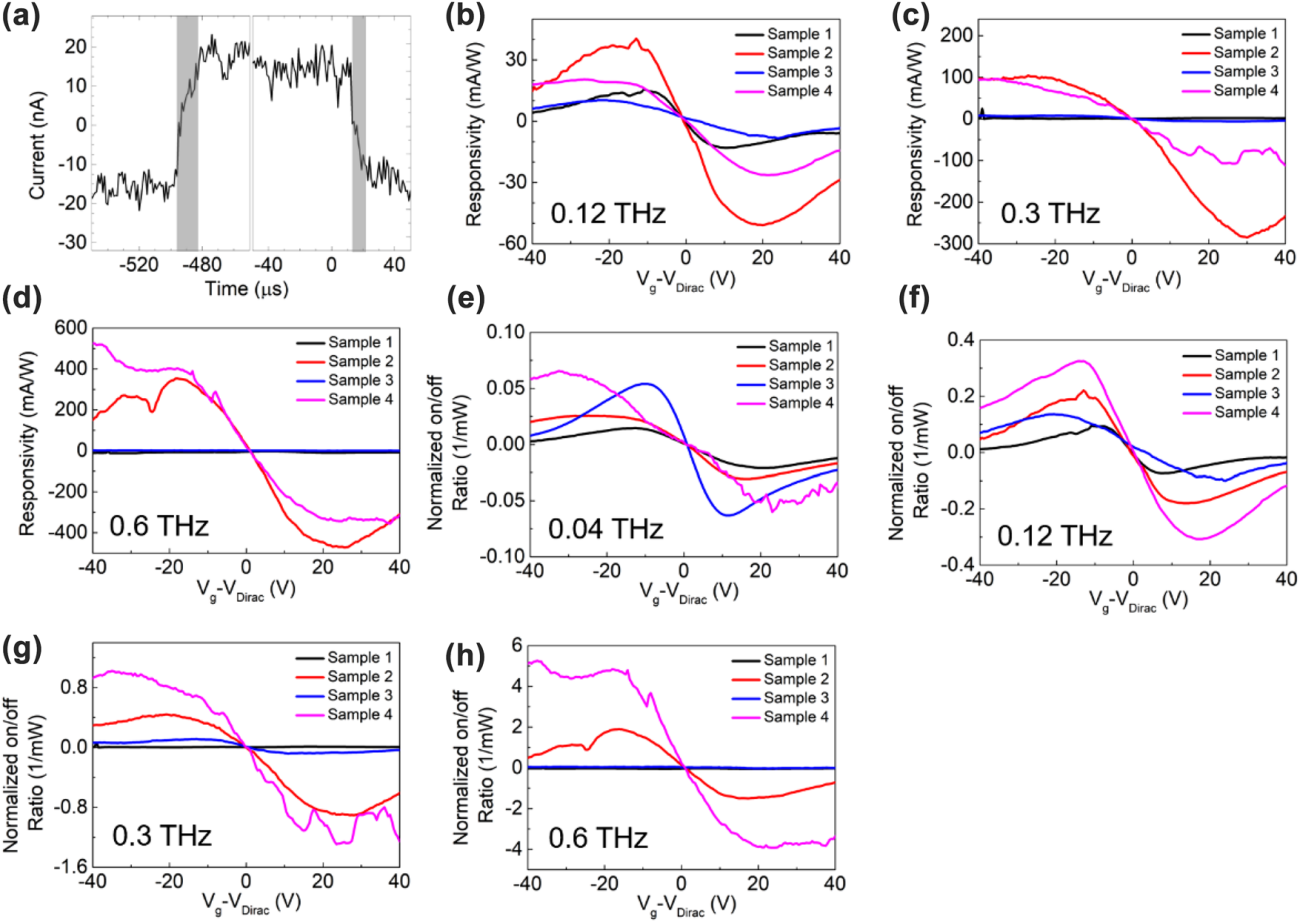
The oscillogram, the responsivity and normalized on/off ratio. (a) The current response as a function of time at 0.04 THz illumination, 0 V gate voltage and 0 V source–drain bias. (b–d) show the responsivity of all devices as a function of gate voltage at (b) 0.12 THz, (c) 0.3 THz and (d) 0.6 THz illumination. (e–h) show the normalized on/off ratio (normalized to the power density) of all devices as a function of gate voltage at (e) 0.04 THz, (f) 0.12 THz, (g) 0.3 THz and (h) 0.6 THz illumination.

However, the response time shown in Figure 4(a) suggests that the plasmon rectification effect cannot be the only contributor. For overdamped plasmons, the energy will be quickly dissipated within nanosecond because *ωτ* << 1 (*ωτ* ≈ 0.003 for 40 GHz), so that the response time should be sub-nanosecond or even sub-picosecond. However, the measured fall-time is about 8 μs, with rise-time of about 16 μs. Such long response time suggests that a slower effect, i.e., the PTE effect, also contributes to the response. Note that the response time is compared between a 30 nm-channel and a 4 μm-channel device in Ref. [21], and the rise-time/fall-time were found to be 0.8 μs/1.5 μs for the former and 15 μs/13 μs for the latter. This means that the response time of PTE detectors is proportional to channel length. Also note that Ref [11] achieved response time of ∼100 ps with 3 μm CVD-grown graphene, whose mobility is under estimated as 1500 cm^2^/Vs. Then it can be estimated that the response time of PTE detectors with 200 μm CVD-grown graphene should be on the scale of 1–10 μs, which is close to the measured 24 μs for our device. Therefore, it can be inferred that PTE effect plays a nontrivial role in our device.

So far, the PTE effect can explain the long response time but fail to explain the polarization-dependence and the peaking profile, while the plasmon rectification effect can explain the polarization-dependence and the peaking profile but fail to explain the long response time. Since it has been indicated in many literature that plasmons will decay into hot carriers [29–31], it is reasonable to infer that graphene plasmons will also decay into hot carriers. Although the mobility of graphene is lower than 2600 cm^2^/Vs after fabrication, overdamped plasmons will decay into hot carriers, and thus enhancing the photo-response. Then the plasmon-enhanced PTE effect can explain both the polarization-dependence and the long rise time very well. Because *ωτ* increases as the incidence frequency increases, the response is anticipated to be higher for incidence with higher frequency. Figure 4(b–d) show that the responsivities of Sample 2 and Sample 4 keep going up as the incidence frequency increases, and the shape is the same as shown in Figure 2(e). The maximum responsivity can exceed 400 mA/W at 0.6 THz and the minimum NEP is about 700 pW/Hz^0.5^. For Sample 1 and Sample 3, however, we only found identifiable response up to 0.12 THz for the former and 0.3 THz for the latter, and no response under 0.6 THz illumination, possibly due to the low output power of the THz source (the power density reduces from 2.5 mW/cm^2^ at 40 GHz to 0.2 mW/cm^2^ at 0.6 THz). For comparison between Sample 2 and Sample 4, however, Sample 4 does not show higher response than Sample 2. Nevertheless, Sample 4 has fewer amounts of graphene ribbons than Sample 2 does. The resistance of Sample 4 (about 1400–2000 Ω) is 2–3 times that of Sample 2 (about 600–900 Ω), so that the on/off ratio is a better indication of enhancement in terahertz response. As the power of the source changes as the frequency changes, the on/off ratio fails in comparing the enhancement in terahertz response under different frequency, and then the on/off ratio is normalized with respect to the received power (the product of the power density and the effective area *S*_eff_ = max{*πλ* ^2^/4, *S*_device_}). As shown in Figure 4(e–h), Sample 4 shows higher normalized on/off ratio than Sample 2 does, especially for higher frequencies. This means that the enhancement on THz absorption is stronger for devices with lower graphene duty cycle. Then it is concluded that THz absorption of graphene plasmons can be enhanced by designing metallic structures, which will finally enhance the orthogonal overdamped plasmon rectification effect and the PTE effect, yielding stronger THz response. These results will be important for raising the responsivity of THz detectors.

## 4 Conclusions

In conclusion, we designed and fabricated THz detectors based on graphene micro-ribbons. Metallic gratings were applied to some of the detectors in order to enhance the excitation efficiency of graphene plasmons. The polarization dependence and the peaking profile of the response suggested that orthogonal overdamped plasmon rectification effect was a main contributor. The polarization dependence and the response time suggested that the plasmon-enhanced PTE effect also contributed. Devices with metallic gratings showed stronger response than devices without metallic gratings. Devices with lower graphene duty cycle showed higher normalized on/off ratio than devices with higher graphene duty cycle, especially at higher frequencies. Then it is concluded that metallic metamaterials can enhance the coupling efficiency between graphene plasmons and THz incidence, especially when the graphene duty cycle is low, thus enhancing the response of THz detectors.

## 5 Experimantal section

### 5.1 Device fabrication

The designed structures were fabricated on a mediumly-doped Si/SiO_2_ substrate. The mediumly-doped silicon promises both back-gate tunability and transmission. Initially, part of SiO_2_ was etched and a back gate electrode of Cr/Au (30/100 nm) was deposited onto the mediumly-doped Si by thermal evaporation. Subsequently, a commercial CVD-grown monolayer graphene (purchased from Nanjing XFNANO Materials Tech Co., Ltd) sheet with pre-fabrication mobility of 3000 cm^2^/Vs was wet-transferred onto the substrate, followed by UV-lithography and O_2_ ion etching to pattern it into rake head shape (y-oriented ribbons with one end connected to an *x*-oriented graphene bar, as shown in Figure 3(a)). Then the source and drain contacts of Cr/Au (20/80 nm) were grown by magnetron sputtering. After that, an ultrathin layer (30 nm) of Al_2_O_3_ was grown by atomic layer deposition. Finally, metallic gratings of Au (80 nm) were grown on the top of the Al_2_O_3_ layer for some of the devices by magnetron sputtering. The devices without the final step are Sample 1 and Sample 3. The devices with the final step are Sample 2 and Sample 4.

### 5.2 Optoelectronic measurement

All optoelectronic measurements were performed at room temperature and ambient environment. 0.04 THz wave was obtained from a microwave source (Agilent E8257D, 20–40 GHz). 0.12 THz and 0.3 THz waves were obtained by equipping the microwave source with VDI multipliers (WR 2.8 and WR 9 Tripler). 0.6 THz was obtained by equipping the microwave source (Agilent E8257D, 9–13 GHz) with a 54× frequency multiplier. The power densities were 2.5 mW/cm^2^ for 0.04 THz, 1 mW/cm^2^ for 0.12 THz and 0.3 THz, and 0.2 mW/cm^2^ for 0.6 THz, respectively. The THz response was measured by using a preamplifier (SR570), a lock-in amplifier (SR830), a high-speed sampling oscilloscope and a Source/Measurement Unit (Agilent B2912A). The mobility of graphene *μ*_gra_ is calculated by *μ*_gra_ = *G*_gra_*L/(*C*_eff_*V_ds_*W_gra_), where *G*_gra_ = d*I*_ds_ */*d*V*_g_ is the transconductance of the device, *L* is the channel length, *C*_eff_ = *ε*_0_ *ε*_ox_ *d*_ox_ is the effective capacitance with *ε*_0_ the vacuum dielectric constant, *ε*_ox_ the relative dielectric constant of SiO_2_, *d*_ox_ the thickness of SiO_2_, *V*_ds_ is the applied source-drain voltage, *W*_gra_ = N*W_rib_ is the total width of graphene channel with *N* the amount of graphene micro-ribbon and *W*_rib_ the width of a single graphene micro-ribbon. The electron density is calculated by *n* = *I*_ds_*L/(*eV*_ds_*μ_gra_*W_gra_) where *I*_ds_ is the measured source-drain current and *e* is the electron charge. Fermi level *E*_f_ of graphene is calculated by *E*_f_ = (*nπ*)^1/2^ *ℏv*_f_, where *ℏ* is the reduced Plank constant and *v*_f_ = *c*/300 is the Fermi velocity. The relaxation time *τ* is calculated by $\tau =\left(\mu_{\text{gra}}E_{\text{f}}\right)/ev_{\text{f}}^{2}$ . The responsivities of the devices are calculated by *I*_res_/(*P*_THz_*S_eff_), where *I*_res_ is the measured response current, *P*_THz_ is the power density of the incidence, *S*_eff_ = max{*S*_dev_, *S*_THz_} is the effective area, with *S*_dev_ = 0.75 mm^2^ (the devices are 1.5 mm long in the *x*-coordinate and 0.5 mm long in the *y*-coordinate) and *S*_THz_ = *πλ* ^2^/4 (*S*_THz_ ≈ 44.18 mm^2^, 4.91 mm^2^, 0.79 mm^2^ and 0.20 mm^2^ for 0.04 THz, 0.12 THz, 0.3 THz and 0.6 THz). The normalized on/off ratio is calculated by *I*_res_/(*I*_dark_*P_THz_*S_eff_). Minimum NEP is calculated by NEP = *i*_n_/*R*, where *i*_n_ = 280 pA/Hz^0.5^ is the measured noise current and *R* = 400 mA/Hz^0.5^ is the maximum responsivity.

## Supplementary Material

Supplementary Material Details
