# Supplementary material for: Graphene plasmons-enhanced terahertz response assisted by metallic gratings
Source: Nanophotonics. 2022 Nov 4;11(21):4737–45. doi: 10.1515/nanoph-2022-0455 (PMC11501250; doi:10.1515/nanoph-2022-0455)
Supplement: Supplementary file 1 — Supplementary Material Details [file j_nanoph-2022-0455_suppl_001.pdf]

Supplementary Information

Figs. S1(a,c) shows the measured current of Sample 1 and Sample 3, respectively, by Agilent B2912A right after fabrication. Figs. S1(b,d) shows the extracted mobility and Fermi level corresponding to Figs. S1(a,c). Figs. S1(e,f) shows the measured current and extracted mobility of Sample 1 and Sample3 by Keithly 4200, six months after fabrication. Fig. S1(g) shows the measured current of Sample 2 right after fabrication, and the inset shows the effective capacitance of the gated region and the ungated region. Fig. S1(h) shows the extracted carrier relaxation time of Sample 1 and Sample 3, right after and six months after fabrication.

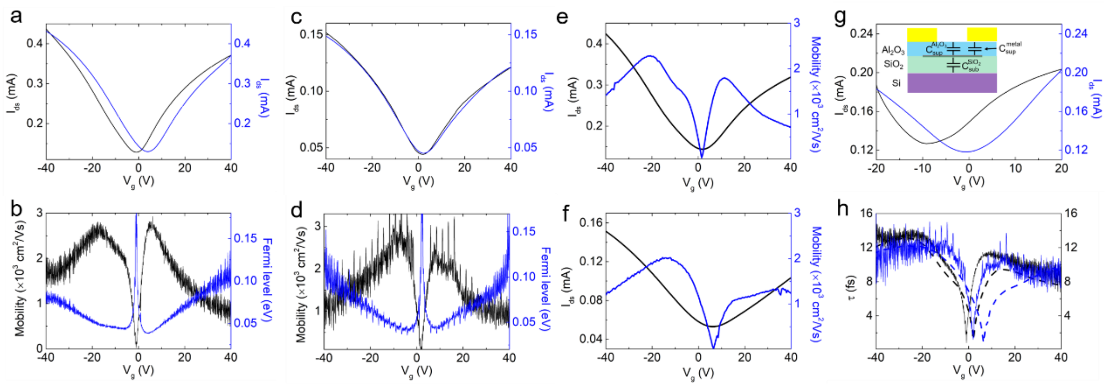

Figure S1 (a) The measured current, (b) mobility (black) and graphene Fermi level (blue) of Sample 1 as a function of back gate voltage right after fabrication. (c) The measured current, (d) mobility (black) and graphene Fermi level (blue) of Sample 3 as a function of back gate voltage right after fabrication. (e) The measured current (black) and mobility (blue) of Sample 1 six months after fabrication. (f) The measured current (black) and mobility (blue) of Sample 3 six months after fabrication. (g) The measured current of Sample 2 right after fabrication. (h) The carrier relaxation time of Sample 1 (black curve) and Sample 3 (blue curve) right after fabrication (solid curve) and six months after fabrication (dashed curve).

Fig. S2(a) schematically shows the tilted Fermi level when crossing the charge neutrality point. There is certain number of electrons/holes at the left/right side of the charge neutrality point. Compared with an ideal condition, in which every single point is on the charge neutrality point, the source-drain current is higher because of the excess electrons or holes, and then the transconductance reduces to ~0 μS (Fig. S2(b)). Consequently, the calculated mobility drops to ~0 cm^2/Vs, while the calculated charge carrier density and the Fermi level reaches a maximum Fig. 2(c).

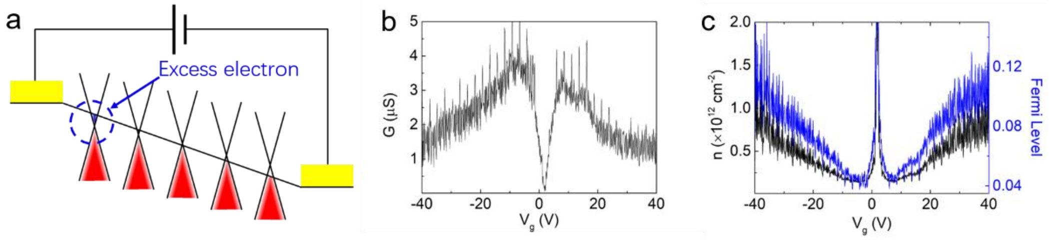

Figure S2 (a) Illustration of the energy band when a negative source-drain bias is applied. (b) The calculated transconductance corresponding to Fig. S1(a). (c) The calculated electron density and Fermi level corresponding to Fig. S1(a).

The solid curves in Fig. S3 shows the measured transmission spectrum by THz time-domain spectroscopy of the bare silicon substrate and the fabricated device Sample 4. The dashed curves in Fig. S3 shows the simulated transmission spectrum by THz time-domain spectroscopy of the bare silicon substrate and the fabricated device Sample 4. In the simulation, mediumly-doped silicon is characterized by the Drude model. The resistivity of the silicon substrate is measured to be ~ 8 Ωcm, and then the dopant density can be estimated to be ~1-3 × 10^{15} cm^{-3}, which corresponds to ω_p ~ 3.5-6 × 10^{12}. By performing the FDTD simulation, we find that the simulation results correspond the best with the measured data by setting $\omega_p = 4.8 \times 10^{12}$ rad and $\gamma = 6.6 \times 10^{12}$ rad. The correspondence between the simulation and the measurement indicates that the devices are well fabricated.

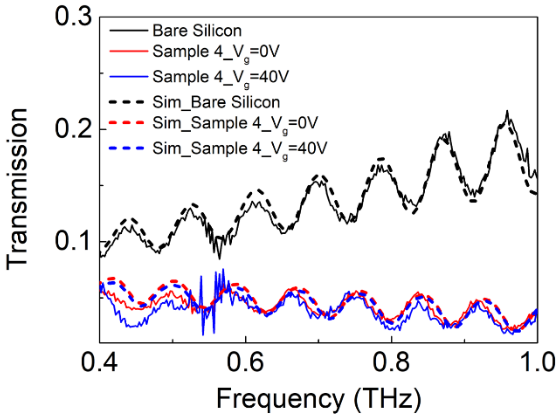

Figure S3 The experimentally measured transmission spectra for the silicon substrate with V_g = 0 V (solid black curve), Sample 4 with V_g = 0 V (solid red curve) and Sample 4 with V_g = 40 V (solid blue curve), and the numerically simulated transmission spectra for the silicon substrate with V_g = 0 V (dashed black curve), Sample 4 with V_g = 0 V (dashed red curve) and Sample 4 with V_g = 40 V (dashed blue curve).
